# Supplementary material for: Multi-Institutional Retrospective Case-Control Study Evaluating Clinical Outcomes of Foals with Small Intestinal Strangulating Obstruction: 2000–2020
Source: Animals (Basel). 2022 May 27;12(11):1374. doi: 10.3390/ani12111374 (PMC9179310; doi:10.3390/ani12111374)
Supplement: Supplementary file 1 [file animals-12-01374-s001.zip › animals-1679068-supplementary.pdf]

Supplementary Table S1. Breeds of foals and adults recovered from surgery.

Breed	Foals recovered (% Short-term survival)	Adults recovered (% short-term survival)
Thoroughbred	9 (92.3)	22 (64.3)
Quarter Horse	1 (33.0)	12 (60.0)
Warmblood	4 (57.1)	10 (90.9)
Arabian	2 (100)	7 (46.2)
Andalusian	2 (66.7)	-
Standardbred	6 (66.7)	7 (100)
Pony	-	2 (66.7)
Morgan	-	2 (20.0)
American Saddlebred	-	2 (50.0)
Tennessee Walking Horse	-	2 (100)
Shire	-	1 (100)
Percheron	1 (100)	1 (100)
Quarab (Arabian-Quarter Horse Cross)	-	1 (100)
Gypsy Cob	-	1 (100)

Supplementary Table S2. Foals recovered from surgery and case-matched adults.

Foal Lesion	Adult Lesion
Intussusception	Strangulating lipoma<br>Strangulating lipoma<br>Strangulating lipoma
Volvulus secondary to ascarid impaction	Volvulus secondary to jejunal impaction<br>Volvulus secondary to ileal impaction<br>Volvulus secondary to jejunal impaction
Ileus and volvulus	Volvulus<br>Volvulus with ileus<br>Strangulating lipoma with volvulus
Umbilical Richter's hernia	Bilateral inguinal hernia<br>Left inguinal hernia<br>Strangulating lipoma
Intussusception	Gastrosplenic ligament entrapment<br>Gastrosplenic ligament entrapment<br>Strangulation lipoma
Segmental volvulus	Gastrosplenic ligament entrapment<br>Strangulating lipoma<br>Strangulating lipoma
Mesenteric volvulus	Mesenteric rent<br>Mesenteric rent<br>Strangulating lipoma
Mesenteric volvulus	Mesenteric volvulus<br>Mesenteric volvulus<br>Strangulating lipoma
Umbilical Richter's hernia	Omental rent<br>Segmental volvulus<br>Herniation through laparoscopic portal site

Small intestinal volvulus	Gastrosplenic ligament entrapment<br>Small intestinal volvulus<br>Small intestinal entrapment in abdominal wall
Volvulus nodosus	Strangulating lipoma<br>Mesenteric root volvulus<br>Segmental volvulus
Segmental volvulus	Mesenteric root volvulus<br>Segmental volvulus<br>Segmental volvulus
Intussusception secondary to intraluminal hamartoma	Adhesions and secondary volvulus<br>Segmental volvulus<br>Left inguinal hernia
Ileal strangulation by mesodiverticular band	Strangulating lipoma<br>Strangulating lipoma<br>Ileal strangulation by nephrosplenic ligament rent
Mesenteric rent	Volvulus secondary to adhesions<br>Volvulus<br>Strangulating lipoma
Volvulus nodosus	Segmental volvulus<br>Segmental volvulus<br>Strangulating lipoma with peritonitis
Ileocecal intussusception	Mesenteric root volvulus<br>Segmental volvulus<br>Mesoduodenal rent
Ischemia secondary to ascarid impaction	Volvulus secondary to omental adhesion to jejunum<br>Segmental volvulus<br>Omental rent with jejunal encarceration
Segmental volvulus	Gastrosplenic ligament entrapment<br>Strangulating lipoma<br>Strangulating lipoma
Segmental volvulus	Omental rent<br>Segmental volvulus<br>Strangulating lipoma
intussusception	Segmental volvulus<br>Strangulating lipoma<br>Intussusception
Segmental volvulus – Necrotizing enterocolitis	Jejunal abscess with adhesion and secondary volvulus<br>Segmental volvulus<br>Strangulating lipoma
Volvulus	Strangulating lipoma<br>Strangulating lipoma<br>Strangulating lipoma
Bilateral inguinal hernias (unreducible left, reducible right)	Left inguinal hernia<br>Left inguinal hernia, traumatic body wall tear<br>Left inguinal hernia
Ruptured inguinal hernia	Left inguinal hernia<br>Right inguinal hernia
